# Identification of Genes Involved in Polysaccharide-Independent *Staphylococcus aureus* Biofilm Formation

**DOI:** 10.1371/journal.pone.0010146

**Published:** 2010-04-14

**Authors:** Blaise R. Boles, Matthew Thoendel, Aleeza J. Roth, Alexander R. Horswill

**Affiliations:** Department of Microbiology, Roy J. and Lucille A. Carver College of Medicine, University of Iowa, Iowa City, Iowa, United States of America; Columbia University, United States of America

## Abstract

*Staphylococcus aureus* is a potent biofilm former on host tissue and medical implants, and biofilm growth is a critical virulence determinant for chronic infections. Recent studies suggest that many clinical isolates form polysaccharide-independent biofilms. However, a systematic screen for defective mutants has not been performed to identify factors important for biofilm formation in these strains. We created a library of 14,880 mariner transposon mutants in a *S. aureus* strain that generates a proteinaceous and extracellular DNA based biofilm matrix. The library was screened for biofilm defects and 31 transposon mutants conferred a reproducible phenotype. In the pool, 16 mutants overproduced extracellular proteases and the protease inhibitor α_2_-macroglobulin restored biofilm capacity to 13 of these mutants. The other 15 mutants in the pool displayed normal protease levels and had defects in genes involved in autolysis, osmoregulation, or uncharacterized membrane proteins. Two transposon mutants of interest in the GraRS two-component system and a putative inositol monophosphatase were confirmed in a flow cell biofilm model, genetically complemented, and further verified in a community-associated methicillin-resistant *S. aureus* (CA-MRSA) isolate. Collectively, our screen for biofilm defective mutants identified novel loci involved in *S. aureus* biofilm formation and underscored the importance of extracellular protease activity and autolysis in biofilm development.

## Introduction

*Staphylococcus aureus* is a human commensal and the causative agent of diverse acute and chronic bacterial infections. The chronic infections persist and cause significant morbidity and mortality to the patient due to the development of a recalcitrant biofilm structure. Compared to the free-living (planktonic) state, *S. aureus* living in biofilms exhibit significant differences in gene expression and physiology [1], and the close proximity of organisms may also allow cooperative metabolic functions, promote horizontal gene transfer, and facilitate cell-to-cell communication [2], [3]. The most notorious biofilm characteristic is their extraordinary resistance to antimicrobial killing [4]. In a recent comparison, we observed a six-log difference in cell viability in the presence of antibiotics of an *S. aureus* biofilm versus planktonic cells [5].

Despite the important role of *S. aureus* biofilms in disease, our understanding of the molecular mechanisms contributing to biofilm formation is incomplete. Recent studies of *S. aureus* biofilm development suggest that the extracellular matrix consists of proteins, DNA, and/or polysaccharide (also called the polysaccharide intercellular adhesin or PIA). In support of this proposal, compounds capable of dissolving matrix components (proteases, DNAse, or glycoside hydrolases) can disrupt established biofilms or prevent the formation of a biofilm [5], [6], [7], [8]. Recently, it has become evident that emerging clinical *S. aureus* isolates are not reliant on PIA for biofilm formation [7], [9], [10]. Protein-mediated biofilm formation has emerged as a prominent alternative to PIA, and many surface adhesins, such as Bap [11], Spa [12], FnBPA and FnBPB [13], and SasG [14], [15], have been implicated in this divergent biofilm mechanism. Biofilms produced by these PIA-independent strains are unaffected by polysaccharide-degrading enzymes, such as dispersin B [6], or mutations in the *ica* gene locus that generates PIA [1], [5], [7], [13]. Therefore, we set out to uncover PIA-independent mechanisms of biofilm formation.

Here we report the generation of a mariner transposon mutant library of 14,880 mutants in a *S. aureus* strain that develops a biofilm by a PIA-independent mechanism. This library was screened for reduced biofilm formation in a microtiter assay and numerous novel loci were identified. This work expands our understanding of genetic factors controlling biofilm formation and may provide potential targets for therapeutic intervention.

## Results

### Identification of mariner transposon mutants defective in biofilm formation

To identify genes involved in PIA-independent biofilm formation, we mutagenized *S. aureus* strain SH1000 using the bursa aurealis (mariner) transposon mutagenesis system [16]. This strain was chosen for mutagenesis and screening because it forms PIA-independent biofilms and is readily amendable to genetic manipulation [5]. Preliminary testing of the mariner transposon system in strain SH1000 was shown to be successful [10]. Altogether, a transposon mutant library of 14,880 mutants was created and banked for further analysis.

Initial screening for biofilm formation in microtiter plates yielded 91 mutants with a defect in attachment. Mutants with severe growth defects were eliminated from further analysis and the remaining potential biofilm mutants were reexamined for reproducibility in the biofilm assay. Thirty-one mutants displayed reproducible biofilm formation defects and arbitrary PCR and sequencing was used to map the insertion location (Table 1). Transposon insertions resulting in a biofilm phenotype were mapped to a variety of genetic loci. Some of the loci, such as *altA*, *fmtA*, *graS*, *rsbU* and *rsbV* have previously been shown to be important in *S. aureus* biofilm formation [10], [17], [18], [19]. The remaining mutants had insertions in genes not previously identified to be involved in biofilm formation.

**Table 1:** Biofilm defective mutants.

Mutant #^a^	8325 ORF^b^	Nucleotide Insertion #	Gene Name^b^	Description^b^	% Biofilm formation^c^
12 D3	2147	2020811		Hypothetical membrane protein	52+/−7
42 F6	19	23385	*purA*	Adenylosuccinate synthetase	51+/−4
46 E1	02667	2452369	*gltT*	proton/sodium-glutamate symport protein	53+/−8
46 H7	718	702698		Hypothetical membrane protein	49+/−5
49 F10	2444	2268603	*opuD*	Osmoprotectant transporter	55+/−4
51 G5	998	970896	*fmtA*	autolysis related protein	31+/−7
52 E4	998	970888	*fmtA*	autolysis related protein	35+/−5
54 C2	2444	2268611	*opuD*	Osmoprotectant transporter	54+/−7
57 B10	998	970904	*fmtA*	autolysis related protein	34+/−4
62 F10	998	970842	*fmtA*	autolysis related protein	49+/−8
79 B5	2000	1910805		glutamate-1-semialdehyde-2,1-aminomutase	66+/−7
123 D2	621	613148		Conserved Hypothetical	52+/−4
128 B10	1430	1368060		Glucose group IIA phosphotransferase protein	61+/−6
130 G5	994	964397	*atlA*	Bifunctional autolysin precursor	34+/−3
132 G7	994	964408	*atlA*	Bifunctional autolysin precursor	31+/−5
3 C5	2301	2133979	*rsbU*	Sigma B locus	26+/−7
19 C7	83	90302	*sbnI*	Siderophore synthesis	55+/−4
37 C9	666	653778	*graS*	membrane sensor histidine kinase	27+/−6
37 G12	2300	2133287	*rsbV*	Sigma B locus	31+/−9
51 F7	1589	1515687		Segregation and condensation protein A or B	54+/−6
52 H3	2418	2247610		Drug resistance transporter	52+/−6
60 G7	1055	1022985	*imp*	Inositol monophosphatase	39+/−8
63 G6	1734	1637360		ATPase family	57+/−7
64 G10	1055	1022984	*imp*	Inositol monophosphatase	35+/−4
83 F3	1721	1624961		Conserved Hypothetical	58+/−9
90 H11	149	160552		N-acetyl-glutamyl-phosphate reductase	61+/−8
114 E5	1066	1034172		Protoheme IX farnesyltransferase	57+/−7
116 G6	1066	1034170		Protoheme IX farnesyltransferase	60+/−8
128 D3	75	80466	*sbnA*	siderophore synthesis	59+/−5
133 H10	2880	2653709		Glycosyltransferase	63+/−6
141 A2	79	84941	*sbnE*	siderophore synthesis	57+/−7

a Order of mutants listed matches the ordering in Figures 1 and 2.

b ORF numbers, gene names, and descriptions are from the National Microbial Pathogen Data Resource web site (www.nmpdr.org).

c Percentages of biofilm formation for each mutant relative to wild type represent the means (+/− standard deviations) from four independent experiments each performed in triplicate.

### Extracellular protease activity of biofilm mutants

Recent studies have demonstrated that protease activity can have antibiofilm effects [5], [7], [20], [21]. To determine if any of the biofilm defective mutants produced increased levels of extracellular protease activity, we assayed protease levels in culture supernatants of the biofilm mutants (Fig. 1). We found that 16 of the 31 transposon mutants displayed an increase in protease activity in cell free supernatants. We hypothesized that the increase in extracellular protease production could be the cause of the biofilm defect in these mutants. To test this hypothesis, the general protease inhibitor α_2_-macroglobulin was added to mutant strains from the beginning of biofilm growth [15]. The addition of this protease inhibitor restored biofilm formation in 13 of the 16 protease overproducing mutants (blue bars, Fig. 2b). Recovery of the *rsbU* and *rsbV* insertions with α_2_-macroglobulin supports our previous report where the biofilm defect of a *sigB* deletion was recovered with the same inhibitor [10]. The three protease overproducing mutants that did not form a biofilm in the presence of α_2_-macroglobulin had insertions in a two-component system (*graS*, mutant 37 C9) or in a probable inositol monophosphate phosphatase (hereafter the gene is called “*imp”*, mutants 60 G7 & 64 G10). In mutants that displayed a wild-type level of extracellular protease activity, the addition of α_2_-macroglobulin had no effect. Collectively, these results indicate that extracellular protease activity is an important contributor to the biofilm defect in 13 of the biofilm mutants.

**Figure 1:**
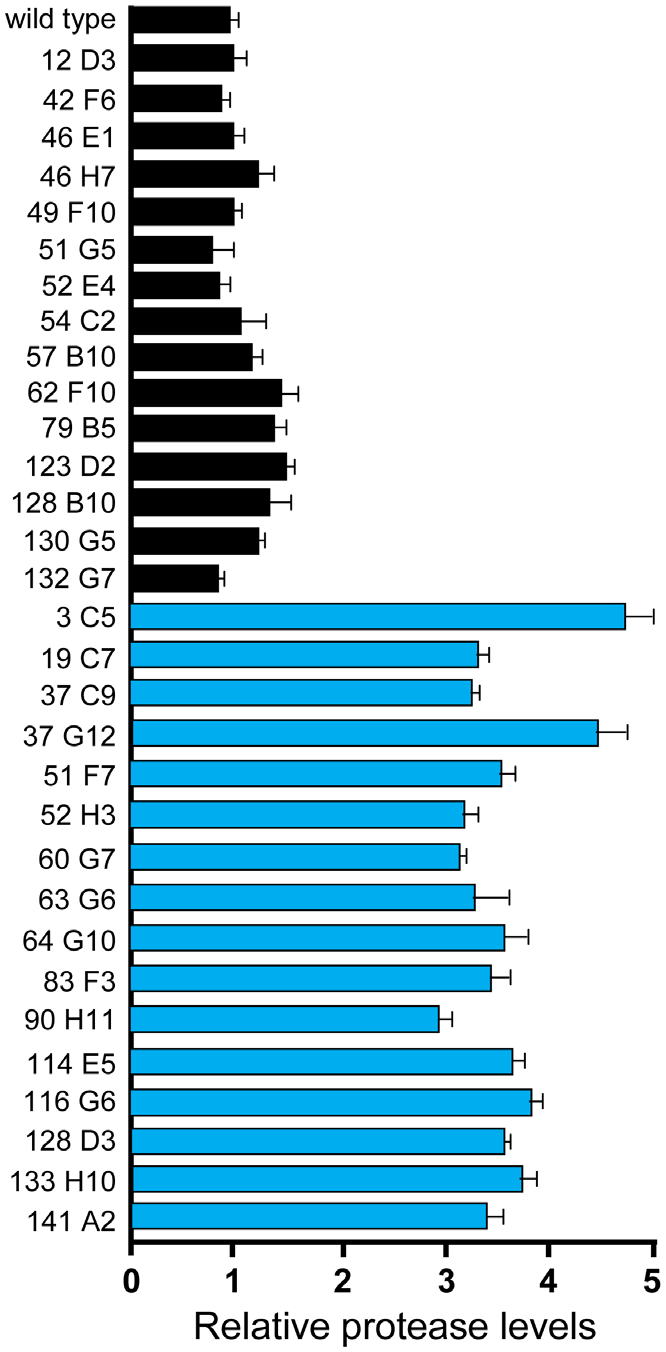
Extracellular protease activity of biofilm mutants. Protease activity in cell-free supernatants from cultures grown in TSB for 12 hours was measured with Azocoll reagent as described in Materials and Methods. Supernatant protease activity of the wild-type strain (SH1000) was set to 1 as a reference. The graph shows the mean of three samples; error bars show standard deviation. Blue bars indicate mutants that show consistently higher levels of protease activity.

**Figure 2:**
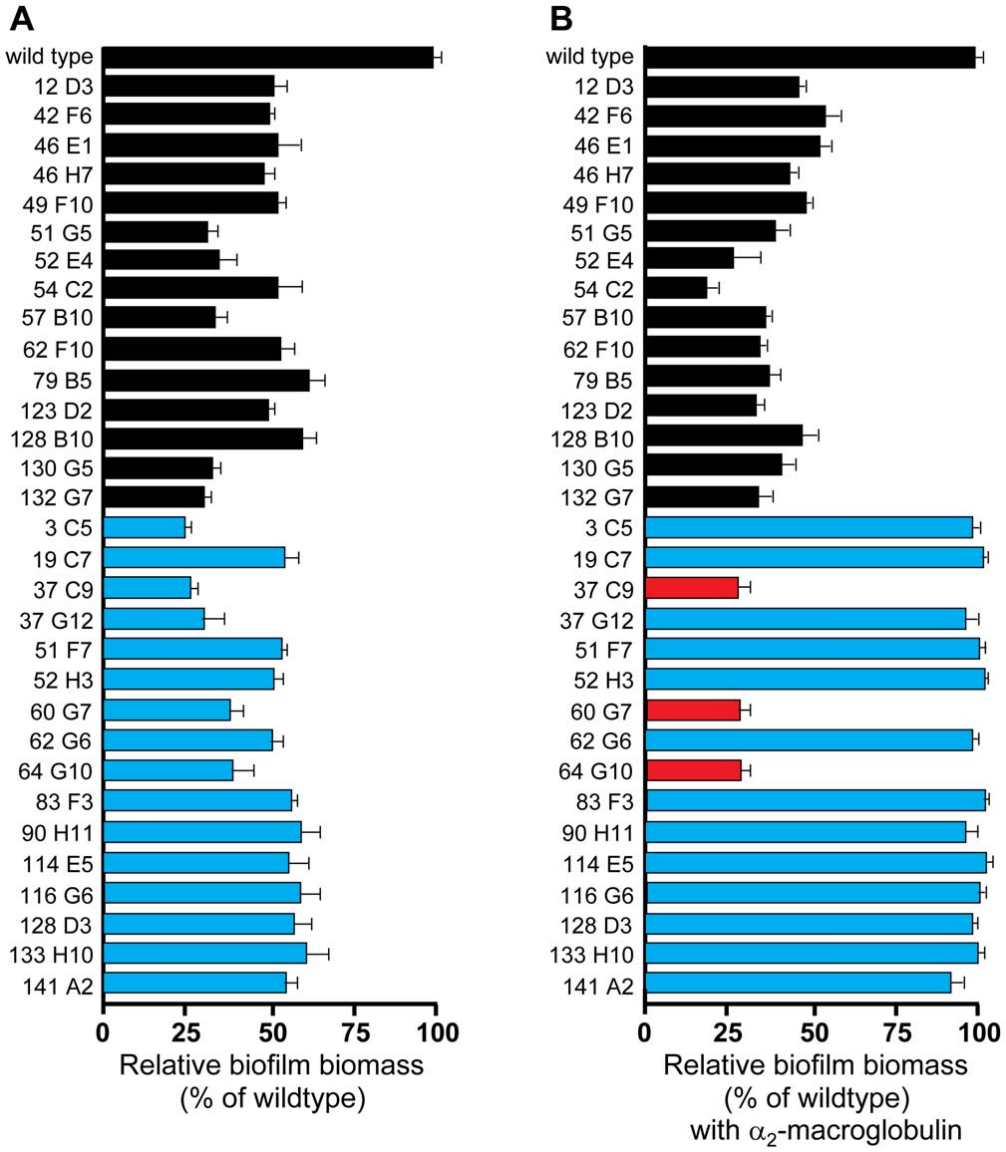
Assessment of biofilm formation in transposon mutants. Microtiter biofilm assays were performed in the absence (A) or presence (B) of the protease inhibitor α_2_-macroglobulin in triplicate, and the percentages of biofilm formation for each mutant relative to that of the wild type are shown. Error bars show standard deviation. Color coding is as follows: black bars indicate mutants with normal extracellular protease activity, blue bars indicate high protease activity and biofilm recovery with α_2_-macroglobulin, and red bars indicate the *graS* (37 C9) and *imp* (60 G7, 64 G10) mutations that did not recover with α_2_-macroglobulin.

### Extracellular nuclease activity of biofilm mutants

**Figure 3:**
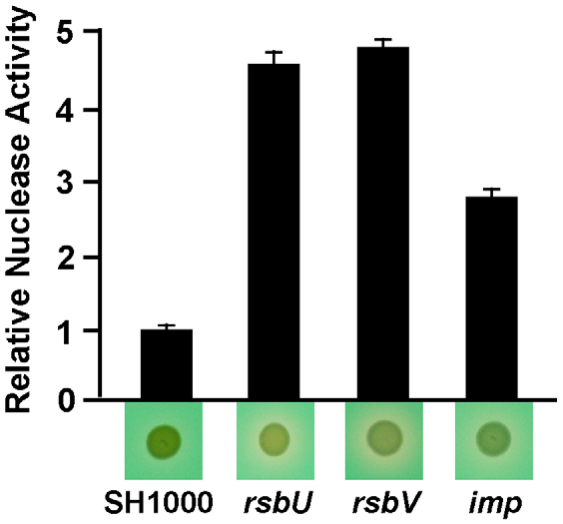
Extracellular nuclease activity of selected biofilm defective mutants. Nuclease activity was measured as described in Materials and Methods and plotted relative to SH1000 levels. For the *imp* mutant, only the results of transposon mutant 60 G7 are shown. Results with *imp* mutant 64 G10 were indistinguishable (data not shown). Underneath the plotted nuclease activity results are representative images of colonies grown on methyl green DNase agar plates. Clearing zones around the colonies is indicative of increased nuclease activity. Error bars show standard deviation.

Evidence in many different bacterial species, including *S. aureus* and CA-MRSA, indicates that extracellular DNA (eDNA) is an important component of the biofilm matrix [6], [8], [9], [22],[23],[24],[25],[26]. Consistent with the key role of eDNA, the expression and extracellular activity of the *S. aureus* thermostable nuclease has profound effects on biofilm maturation [6], [8]. Based on these results, we hypothesized that some mutants may be unable to form biofilms due to increased nuclease expression. To address this hypothesis, a phenotypic plate assay was employed to assess the levels of extracellular nuclease activity in biofilm defective mutants. After testing all 31 mutants, four mutants in the pool, 3 C5 (*rsbU*), 37 G12 (*rsbV*), 60 G7 (*imp*) and 64 G10 (*imp*), had increased levels of extracellular nuclease activity (Fig. 3). These four mutants were also tested using a quantitative nuclease assay, and the results confirmed the plate assay phenotypes (Fig. 3). The increased nuclease activity of the *rsbU* and *rsbV* mutants is consistent with microarray profiling of Sigma B defective strains [27].

### Autolysis levels of biofilm mutants

**Figure 4:**
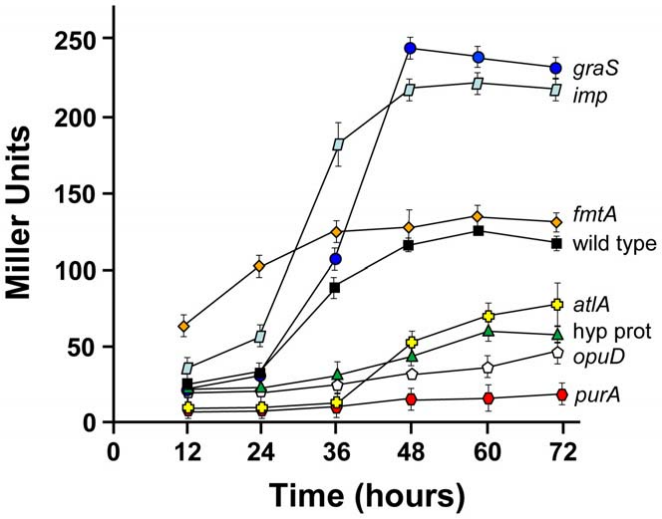
Time course of autolysis in selected biofilm mutants. Planktonic cultures of seven transposon mutants harboring plasmid pAJ22, which expresses cytoplasmic β-galactosidase, were grown for 72 hrs. Every 12 hr during the time course samples were removed from cultures and β-galactosidase activity in cell free supernatants was measured (reported in Miller units). SH1000 with pAJ22 was included as a control (shown as black squares). Results shown were the average of two independent experiments done in triplicate and error bars show standard deviation.

*S. aureus* biofilm matrix is composed in part by eDNA that has been released by autolysis [8], [23]. Therefore if lysis is altered, the change could reduce the ability of *S. aureus* to effectively form a biofilm. To test for this phenotype, we utilized an assay that measures autolysis as a function of β-galactosidase release into culture supernatants [23]. We focused on the 18 transposon mutants in the pool of 31 unable to form a biofilm in the presence of α_2_-macroglobulin (black and red bars, Fig. 2). Due to some genes with multiple insertions (Table 1), this pool was reduced to 12 insertions in unique loci and each of these mutants was transformed with plasmid pAJ22, which constitutively expresses the *lacZ* gene. Cell free culture supernatants were assayed for β-galactosidase activity at indicated time points. During a time course, the autolysis profile of five mutants mirrored wild type (data not shown), while seven of the 12 mutants showed significant changes in β-galactosidase activity (Fig. 4). Four of these seven mutants, 42 F6 (*purA*), 54 C2 (*opuD*), 123 D2 (hypothetical membrane protein), and 132 G7 (*atlA*), all demonstrated reduced autolysis compared to their isogenic parent. Conversely, three mutants 51G5 (*fmtA*), 37 C9 (*graS*), and 60 G7 (*imp*), displayed an increase in autolysis. Of these, the *fmtA* mutant showed high levels of autolysis early in the time course (12–36 hrs, Fig. 4), while lysis in the *graS* and *imp* mutants was more pronounced later in the time course (36–72 hrs, Fig. 4). The altered ability of these mutants to precisely control autolysis (and thus release of eDNA) may contribute to their inability to effectively form biofilms.

### graS and imp mutants are biofilm defective in a CA-MRSA strain

**Figure 5:**
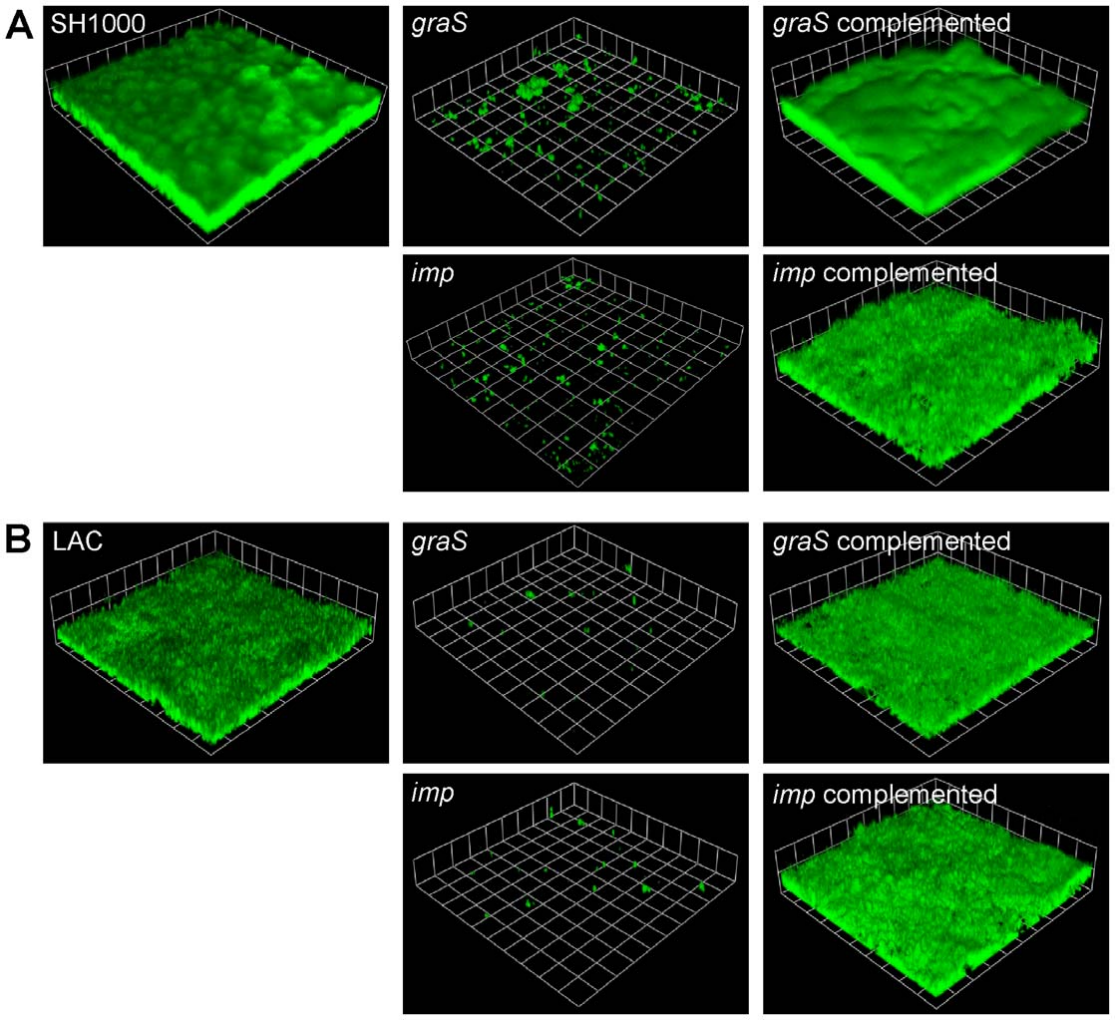
Flow cell biofilm formation of *graS* and *imp* mutants in different strain backgrounds. *S. aureus* strains SH1000 (A) and LAC* (B), and *graS* and *imp* mutations in each strain, were grown in a flow cell apparatus for two days. A *z* series of images was obtained with CLSM, reconstructed with Volocity software, and each side of a grid square is 20 µm in the image reconstruction. The addition of a complementing plasmid containing either the *graRS* or *imp* genes is shown in the last column.

Mutations in the *graS* and *imp* genes were chosen for further study because of their unique combination of autolysis, nuclease, and protease phenotypes (Figs. 2, 3, 4). To assess the biofilm maturation phenotypes in a flowing environment, the *graS* and *imp* transposon mutants were grown in a flow cell biofilm model. As shown in Figure 5A, both the *graS* mutant (37 C9) and the *imp* mutant (60 G7) displayed profound defects in biofilm maturation in the flow cell. Importantly, the biofilm phenotype could be complemented by expressing the wild-type gene from a plasmid.

To address potential issues of strain-to-strain variation, the *graS* and *imp* transposon insertions were transduced into a CA-MRSA isolate called LAC* (erythromycin sensitive variant of LAC, see Materials and Methods). The LAC* strain is a member of the USA300 lineage [28], and previous studies have demonstrated that the strain is a robust biofilm former on diverse surfaces [9], [10]. The *graS* and *imp* insertions were transduced into the LAC* strain and growth in a flow cell biofilm model was compared to wild type (Fig. 5B). Similar to the SH1000 genetic background, the LAC* *graS* and *imp* mutants were defective in biofilm maturation, and again the biofilm phenotype could be complemented through plasmid expression. These results demonstrate that the *graS* and *imp* mutant biofilm phenotypes are consistent across multiple *in vitro* models of biofilm maturation and the phenotype is conserved in a clinical isolate.

## Discussion

The ability of *S. aureus* to form biofilms is an important virulence factor in many persistent infections [3], [29]. Recently, it has been shown that some *S. aureus* clinical isolates do not require polysaccharide production to form a biofilm [1], [7], [9], [10]. To the best of our knowledge, this type of polysaccharide-independent biofilm (termed PIA-independent) has not been systematically investigated through transposon mutagenesis. We created and screened a mariner transposon mutant library in a PIA-independent strain and identified 31 insertions that displayed reproducible biofilm defects. Our screen was large but not saturating, as some loci known to be biofilm deficient in the SH1000 background, such as *sarA* and *dltA*, were not identified (data not shown).

To understand the 31 identified biofilm mutants in more detail, various aspects of biofilm matrix production and breakdown were investigated. With the known importance of proteinaceous material in the matrix [5], [6], [7], we examined the contribution of secreted proteases to the biofilm phenotype. Almost half of the mutant pool displayed an increased level of protease activity in culture supernatants (Fig. 1), and the biofilm phenotype of this subset could be mostly restored with the general inhibitor α_2_-macroglobulin (Fig. 2). This finding suggests a critical and perhaps underappreciated role of the extracellular proteases in biofilm maturation. In recent reports, high levels of protease activity have been linked to biofilm phenotypes in *sigB* and *sarA* mutants [10], [20], [21], and this activity has an additional role in biofilm dispersal [5]. Numerous potential targets for the proteases have been investigated, including surface adhesins BAP [21], [30], Spa [12], SasG [14], [15], FnBPA, and FnBPB [13], and the murein hydrolyases have also been suggested as a target [10].

Along with proteins in the *S. aureus* biofilm matrix, growing evidence indicates eDNA is an important matrix material [8], [9], [23]. Microarray data has indicated that the *nuc* gene is down-regulated during biofilm formation as compared to planktonic cultures [1], suggesting a disregulation of nuclease could lead to defects in biofilm formation. Transposon insertions in *rsbU*, *rsbV*, and *imp* genes resulted in increased nuclease activity in culture supernatants (Fig. 3). Insertions in *rsbU* and *rsbV* eliminate SigB activity [10], and *sigB* mutants are known to have increased *nuc* gene expression [27]. However, the significance of this phenotype is not clear as higher levels of protease activity have been linked to the biofilm phenotype [10], which is supported by the recovery of the *rsbU* and *rsbV* mutants with α_2_-macroglobulin (Fig. 2). Increased nuclease activity may be a factor in the *imp* mutant biofilm phenotype, but further analysis will be necessary to demonstrate a connection.

In order to release eDNA into the environment, *S. aureus* cells lyse and are able to incorporate the eDNA into the biofilm matrix [8], [23]. This autolysis event is a complex and tightly controlled process that involves regulation of holins, antiholins and murein hydrolase activity [31]. In the pool of biofilm-defective mutants, seven displayed altered autolysis levels compared to the isogenic parent. Of this subset, four displayed a reduction in autolysis activity, suggesting they failed to release adequate amounts of eDNA as biofilm matrix material. As anticipated, mutations in the major murein hydrolase, *atlA*, possessed low levels of autolytic activity. Mutants in *atlA* in *S. aureus* and *Staphylococcus epidermidis* (called *atlE*) have previously been shown to have defects in biofilm formation [17], [32], and the expression of *atlA* is known to increase during biofilm growth [33]. In *S. epidermidis*, the reduction in biofilm formation by *atlE* mutants has been attributed to the ability of this protein to bind vitronectin, an abundant glycoprotein found in serum [32]. Considering that no serum was present in our biofilm growth conditions, we speculate that the biofilm defect in *S. aureus atlA* mutants is due to decreased autolysis and DNA release.

Three other transposon insertions in an osmoprotectant transporter (OpuD homolog), *purA*, and a hypothetical membrane protein (mutant 123 D2) also displayed decreased autolysis. The OpuD-like protein has sequence similarity to a glycine-betaine osmoprotectant transporter that is necessary for biofilm formation in *Vibrio cholerae* [34]. The *purA* gene encodes for a putative adenylosuccinate synthetase that is upregulated during biofilm growth [1], and the *purA* knockout displayed the lowest level of autolysis of tested mutants (Fig. 4). Studies in *Bacillus cereus* revealed that a *purA* mutant is unable to effectively form biofilms or release eDNA, and the expression of *purA* is upregulated in a biofilm versus planktonically grown cells [35].

In contrast, transposon insertions in the genes *fmtA*, *graS*, and *imp* displayed an overall increase in autolysis (Fig. 4). FmtA is an autolysis and methicillin-resistance related protein that was identified in screens for increased oxacillin sensitivity in the presence of detergent [36]. Biochemical analysis demonstrated that FmtA is a penicillin-binding protein that is resistant to β-lactam inactivation [37], and the protein is known to localize to the membrane and affect cell wall structure [38]. A previous transposon screen for biofilm defective mutants in a PIA-dependent strain identified the *fmtA* gene three different times [18], suggesting that the FmtA protein is important across both PIA-dependent and independent mechanisms of biofilm formation. The observation that both mutants that decrease autolysis (*altA, opuD, purA*, 123 D2) and increase autolysis (*fmtA, graS, imp*) have biofilm defects suggests that the timing of autolysis and the accumulation of eDNA in the biofilm matrix may be critical in biofilm development.

A transposon insertion in *graS* of the *graRS* (glycopeptide resistance-associated) two-component regulatory system also displayed an increase in autolysis (Fig. 4) and a pronounced biofilm defect in both laboratory and CA-MRSA strains (Fig. 5). Mutations in *graRS* are known to make *S. aureus* hyper-susceptible to vancomycin [39], cationic antimicrobial peptides [40], [41], and cell lysis inducing agents [19], [39], findings which correlate with our observed increase in *graS* mutant autolysis. Genes encoding proteins for D-alanylation of teichoic acids (*dlt* operon) and *atlA* are induced by the GraRS system, and *graRS* mutants are reported to have a microtiter-based biofilm defect [19]. The increase in *graS* mutant autolysis over wild-type levels did not occur until late in the time course (>24 hrs, Fig. 4), with the most significant increase after 36 hrs. In the flow cell biofilms (Fig. 5), most *S. aureus* attachment and preliminary biofilm formation is occurring in the first 24 hr, suggesting that the reduced *dlt* and *atlA* expression are the major contributors to the *graS* mutant biofilm phenotype.

The final mutant that displayed increased autolysis was a transposon insertion in the *imp* gene, which is predicted to encode for an inositol monophosphatase, an enzyme capable of dephosphorylating inositol phosphate to inositol. Mutations in *imp* displayed nuclease overproduction (Fig. 3) and a pronounced biofilm defect (Fig. 5) that was conserved in a CA-MRSA strain. Inositol is an important signaling molecule in eukaryotes, but its role in bacteria is not well understood [42]. The altered nuclease and autolysis levels suggest a potential regulatory role for the *imp* gene in *S. aureus*. Interestingly, an inositol monophosphatase mutant (*suhB*) was found in a search for biofilm defective mutants in *Burkholderia cepacia* [*43*], suggesting this gene may play an important role in biofilm formation in diverse bacterial species. Further analysis will be necessary to define the role of the *imp* gene in biofilm maturation and determine the significance of the observed nuclease and autolysis phenotypes.

Five remaining mutants did not fall into the categories of displaying increased protease or nuclease activity, or having an altered autolysis phenotype. The inactivated genes encode two potential membrane proteins (mutants 12 D3 and 46 H7), a glutamate transporter (*gltT*), a glutamate-1-semialdehyde-2,1-aminomutase, and a glucose group IIA phosphotransferase protein. Microarray studies have demonstrated an upregulation of expression in *gltT* during PIA-dependent biofilm growth, potential evidence of a link between GltT and biofilms [44]. At this time the roles for these five genes and their encoded proteins in biofilm formation is unknown.

Overall, the work presented here confirms published reports and provides new insight into genetic loci required for PIA-independent biofilm formation. Over half of the biofilm defective mutants expressed high levels of extracellular protease activity, and a significant portion of the mutant pool either overproduced nuclease or had altered cell lysis phenotypes, indicating a major biofilm role for proteins and eDNA in the absence of exopolysaccharide. Moving forward, it will be important to assess how these various identified factors collaborate during the biofilm maturation mechanism.

## Materials and Methods

### Strains and growth conditions

The bacterial strains used in this study are described in Tables 1 and 2. Strains of *Escherichia coli* were grown in Luria-Bertani broth or Luria agar plates, and growth medium was supplemented with ampicillin (100 µg/ml) or chloramphenicol (10 µg/ml) as needed for maintenance of plasmids. Strains of *S. aureus* were grown in tryptic soy broth (TSB) or tryptic soy agar (TSA). For selection of chromosomal markers or maintenance of plasmids, *S. aureus* antibiotic concentrations were (in µg/ml) the following: chloramphenicol (Cam), 10; erythromycin (Erm), 10; and tetracycline (Tet), 5. All reagents were purchased from Fisher Scientific (Pittsburg, PA) and Sigma (St. Louis, MO) unless otherwise indicated.

**Table 2:** Strains and plasmids.

Strain or plasmid	Description	Source or reference
Strains		
*E. coli* strains		
DH5α-E	Cloning strain	Invitrogen
BW25141	Cloning strain	[46]
*S. aureus* strains		
SH1000	*sigB^+^* derivative of NCTC8325-4	[56]
LAC	CA-MRSA USA300-0114	[28]
AH1263 (LAC*)	Erm-sensitive LAC	This study
Plasmids		
pAJ22	β-galactosidase expression plasmid	[23]
pBursa	Mariner hopping plasmid	[16]
pFA545	Mariner transposase	[16]
pCm1	*S. aureus* expression plasmid	This study
pCm1-*imp*	*imp* gene cloned into pCm1	This study
pCm1-*graRS*	*graRS* genes cloned into pCm1	This study

Strain LAC was made Erm sensitive by serial passage in TSB in order to cure the strain of the native plasmid pUSA03 that confers Erm resistance [45]. A single colony was picked and saved as Erm sensitive strain AH1263, and hereafter this strain will be referred to as LAC^*^.

### Recombinant DNA and genetic techniques

Restriction and modification enzymes were purchased from New England Biolabs (Beverly, MA). All DNA manipulations were performed in *E. coli* strain BW25141 [46]. Oligonucleotides were synthesized at Integrated DNA Technologies (Coralville, IA). Nonradioactive sequencing was performed at the DNA sequencing facility at the University of Iowa. Plasmids were transformed into *S. aureus* RN4220 by electroporation and moved to other strains using transduction by bacteriophage 80α as described previously [47], [48]. Chromosomal markers were moved between *S. aureus* strains using bacteriophage 80α or 11 transduction.

### Generation and mapping of transposon mutants

SH1000 was transformed with plasmids pFA545 and pBursa, and mutagenesis was performed as described previously [10]. Transposon mutants with secondary *agr* defects were removed from the pool by testing for AIP-I production as described previously [49]. Mutants were banked in deep-well microtiter titer plates in TSB with 10% glycerol and stored at –80°C. Transposon insertion sites were mapped using arbitrary PCR [10].

### Biofilm assays

Microtiter plate biofilms and flow cell biofilms were grown as described previously [5]. For culture media, microtiter biofilms were grown in 66% TSB supplemented with 0.2% glucose, and flow cell biofilms were grown in 2% TSB supplemented with 0.2% glucose. For protease inhibition in microtiter biofilms, cells were added to the plate with α_2_-macroglobulin (final concentration, 0.25 units/ml; Roche). Confocal scanning laser microscopy (CSLM) and image analysis was performed as described previously [5]. Biofilms were treated with 330 nM Syto9 (LIVE/DEAD BacLight Bacterial Viability Kit; Invitrogen, Carlsbad, CA) 15 min prior to visualization.

### Protease and nuclease assays

Quantitative protease activity measurements were determined using Azocoll (Calbiochem, San Diego, CA) reagent as described previously [50]. Difco DNase test agar with methyl green (BD, Franklin Lakes, NJ) was used to screen biofilm mutants for altered nuclease production. Nuclease activity in culture supernatants was measured as described by Heins *et. al* [51].

### Autolysis assay

To determine autolysis activity, overnight cultures of each strain harboring plasmid pAJ22 [52], were diluted to an OD at 600 nm of 0.1 in TSB (no antibiotic) and grown at 37°C shaking at 200 rpm. At various time points, supernatants from each culture were harvested by centrifugation. β-galactosidase activity in the supernatants was determined as described [53] using o-nitrophenyl-beta-d-galactopyranoside as the substrate and reported in Miller units [54].

### Plasmid construction

#### pCM1

Plasmid pCM1 was created to serve as a new chloramphenicol resistant *S. aureus* expression vector. This plasmid was constructed by moving the chloramphenicol acetyltransferase gene into plasmid pAH15 [55] and eliminating erythromycin resistance. The chloramphenicol acetyltransferase gene was amplified using oligonucleotides CLM329: GTTGTT*GCTCAGG*TAAAGGAGGCATATCAAATGAAC and CLM330: GTTGTT*TGATCA*TTATAAAAGCCAGTCATTAGGCCTATC and plasmid pAH5 [55] as template. The PCR product was digested with BclI and Bpu10I and ligated to pAH15 digested with the same enzymes to create pCM1.

#### pCM1-imp

Plasmid pCM1-imp was created by cloning a 1081 base pair PCR product containing the *imp* gene and promoter region into pCM1 that had been restriction digested with HindIII and EcoRI. The oligonucleotides used to generate the *imp* PCR product were: inositolREVecoRI (5′-GAGGAATTCACTGGTTTTATATTGGCGCGTG-3′) and inositiolFORhindIII (5′-GAGAAGCTTTAGfAGTACCTCCTGTATAGTGT-3′). The resulting pCM1-*imp* plasmid has the *imp* gene with its native promoter controlling expression.

#### pCM1-graRS

Plasmid pCM1-*graRS* was created by cloning a 1751 base pair PCR product containing the *graRS* genes without their native promoter into pCM1 that had been restriction digested with KpnI and EcoRI. The oligonucleotides used to generate the *graRS* PCR product were: graSkpn (5′-AAAAAAGGTACCGTTTAAAATGACAAATTTGTC-3′) and graRecoR1 (5′-AAAGAATTCTGATATTGGGTGATATGGATGC-3′). The resulting pCM1-*graRS* plasmid has the *graRS* genes with their expression driven by the *sarA* promoter located on pCM1.
